# Preface

**DOI:** 10.1155/DTE.1.v

**Published:** 1994

**Authors:** Denise A. Cortese

**Affiliations:** Mayo Medical School Mayo Clinic Jacksonville Florida USA


					Diagnostic and Therapeutic Endoscopy, Vol. 1, p. v
Repdnts available directly from the publisher
Photocopying permitted by license only
(C) 1994 Harwood Academic Publishers GmbH
Printed in Malaysia
Preface
Professor Harubumi Kato and Harwood Academic Publishers should be complimented for their work in prepar-
ing the first issue of a new medical journal. This is a task of major proportion at any time. But at this particular
time in the world economy to introduce a medical journal on an international scale devoted to the broad field of
endoscopy takes a special kind of courage, vision, and confidence in the future of international medicine.
The medical economic pressures are such that there is less interest now than in recent years in the areas of tech-
nology development, technology transfer to the clinic setting, and assessment of new technology for clinical ef-
fectiveness. In the rush to reduce the overall costs of medical care in our countries there is a tendency for
administrative policy makers to agree to pay for only those modalities of proven effectiveness. They often accept
the self serving assumption that if a modality is not proven to be effective then it must be ineffective. Medical pol-
icy crafted with this premise in mind will ultimately stifle medical innovation. Worse, it has the potential to en-
courage physicians to overlook new modalities that may prove to be more effective and efficient (cost effective)
that currently approved (paid for) modalities.
Professor Kato's vision ofthe future of medicine goes beyond this. He sees the need and the opportunity for physi-
cians to participate in the continued introduction of new concepts, techniques, and technology into the man-
agement of their patients. He sees doing all this in an atmosphere or medical inquiry to establish the role in
medical practice, if any, of these new modalities. The finding that a new, and often expensive, modality has noth-
ing new to contribute is often just as important as finding a new and effective one.
The journal of DIAGNOSTIC AND THERAPEUTIC ENDOSCOPY is introduced in this spirit. It is dedicated to the
entire field under one journal. There is geographical representation among the editorial boards from Asia, Europe,
and America to facilitate publication of papers from throughout the world. The journal will fulfill a mission by
providing a much needed mechanism for the international exchange of ideas about the ever expanding reach of
diagnostic and therapeutic endoscopes and endoscopic technique. It will fulfill an equally important mission as
it helps establish the appropriate role of these modalities in the future of medical practice.
Congratulations for a fine job to Professor Kato and Harwood Academic Publishers. It is up to the rest of us to
help make the journal a success story in the next several years.
Denise A. Cortese, MD
Professor of Medicine and Thoracic Diseases
Mayo Medical School
Mayo Clinic, Jacksonville, Florida, USA

				

